# Brodie's Abscesses Can Stimulate the Growth Plate in Children

**DOI:** 10.7150/jbji.37266

**Published:** 2019-11-06

**Authors:** Patricia Garcia Pulido, Andrew Hotchen, Ashwin Gojanur, James Corbett, Kuldeep K Stohr

**Affiliations:** Cambridge University Hospital, United Kingdom

## Abstract

**Introduction:** We present a series of children with lower limb Brodie's abscesses (subacute osteomyelitis) with subsequent deformities.

**Method:** A retrospective examination of the paediatric bone and joint infection database from 2014-2017 was performed. All children have MRI scans and blood tests including full blood count, ESR and CRP. MRI identified collections were drained surgically.

**Results:** There were 68 children with bone and joint infections, and 6 had a Brodie's abscess. 4 Brodie's abscesses were adjacent to a growth plate, all these had resultant growth deformities. Some deformities develop up to 3 years after initial presentation.

**Discussion:** We recommend long-term vigilance for growth deformity after a Brodie's abscess. In particular we demonstrate that infection can result in stimulation of the physis, as opposed to growth retardation as generally accepted.

## Introduction

Sub-acute osteomyelitis describes a small subset of osteomyelitis which has milder clinical features than the acute form. Patients typically have a delayed presentation with milder symptoms of fever, systemic upset and pain. A Brodie's abscess occurs in sub-acute osteomyelitis and is a low-grade pyogenic abscess usually with a sclerotic or fibrous margin. Brodie first described chronic tibial abscesses in 1832 during the pre-imaging and pre-antibiotic era^1^. Inflammatory markers characteristically are only mildly elevated on initial presentation. Differentiation from alternative diagnoses such as osteoid osteoma or a stress fracture can prove difficult.

We routinely follow-up our paediatric musculoskeletal infection cases for 2-5 years and have found that despite the milder presentation of subacute osteomyelitis there are nevertheless potential long-term growth effects.

Sub-acute osteomyelitis commonly occurs in the metaphysis adjacent to the physis, however it can also occur in the epiphysis or diaphysis of long bones, it can also occur in bones without a longitudinal physis such as the talus or vertebral body. Brodie's abscesses usually occur in the metaphysis of the bone, subacute osteomyelitis in the diaphysis does not characteristically present with a collection^2^.

The true incidence of Brodie's abscesses is not known but the incidence of both acute and sub-acute osteomyelitis has steadily decreased over the last century^3^-^5^. The decline may be due to the introduction of antibiotic therapy, increased health of children, improved imaging techniques, the introduction of *Haemophilus influenza type B* vaccination^6^ and reduced virulence of the organisms.

## Method

We retrospectively reviewed a database of children admitted with a bone and joint infection over a period of 3 years in our institution (2014-2017). Our institution is a university teaching hospital and a regional centre for paediatric surgery in the South East of the United Kingdom. Over that period a total of 68 children were recorded. Patients routinely have MRI scanning in addition to blood cultures, full blood count, creatinine, electrolytes, liver function tests, C-reactive protein and erythrocyte sedimentation rate. We operate only to debride collections - either intra-osseous or intra-articular, and all children receive intravenous antibiotics for 4-6 weeks.

All patients with any bone and joint infection in the lower limbs are reviewed yearly or 2 - yearly for 5 years with long leg standing limb alignment views as subtle differences may otherwise be missed. Patients are discharged if there is no growth deformity after five years.

### Findings

**Figure 1:**
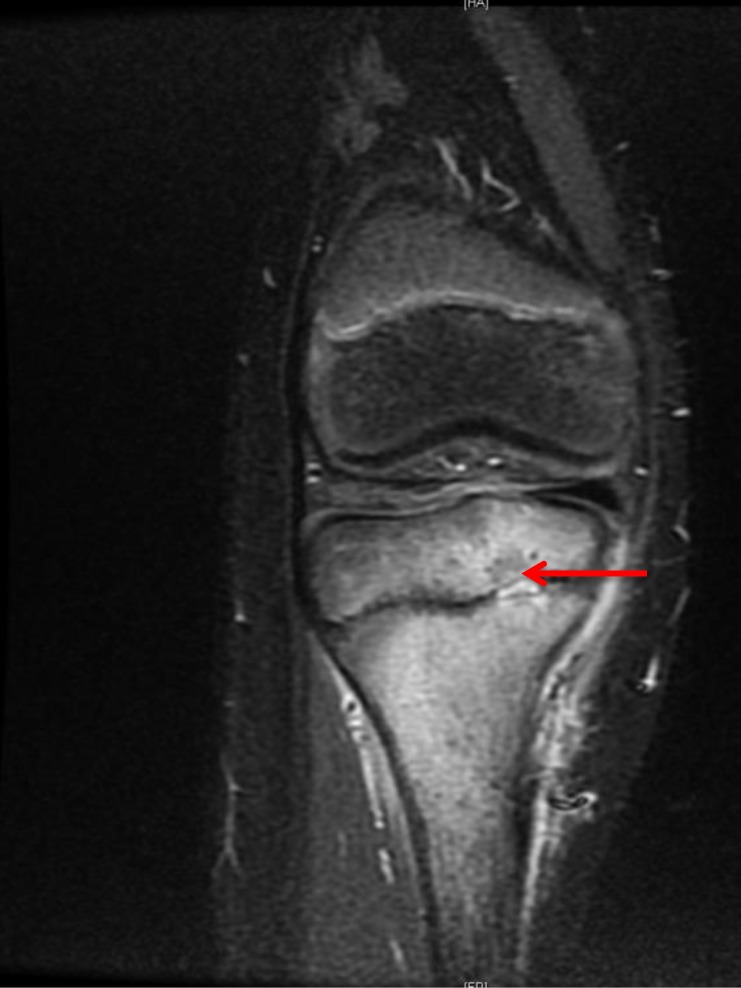
Medial tibial subacute osteomyelitis with a small intraphyseal Brodie's abscess *(marked by red arrow)*.

6 children had a diagnosis of Brodie's abscess. There were 5 boys and 1 girl with a mean age of 3.5 years (range 1-12yr). Three abscesses were juxta-physeal (adjacent to the growth plate the knee joint), and one was intra-physeal *(see Figure 1)*, one abscess was in the femoral neck and one was in the talus. The abscesses in the femoral neck and talus were not close to a physis and neither demonstrated any growth deformity. *Table 1* summarises the four juxta-physeal infections. In our series the infection was predominantly on the metaphyseal side, but extended across the physis in one case. Staphylococcus aureus was identified in 50% of the knee cases. The incidence of Brodie's abscesses in our unit is 9% of all paediatric bone and joint infections.

**Figure 2:**
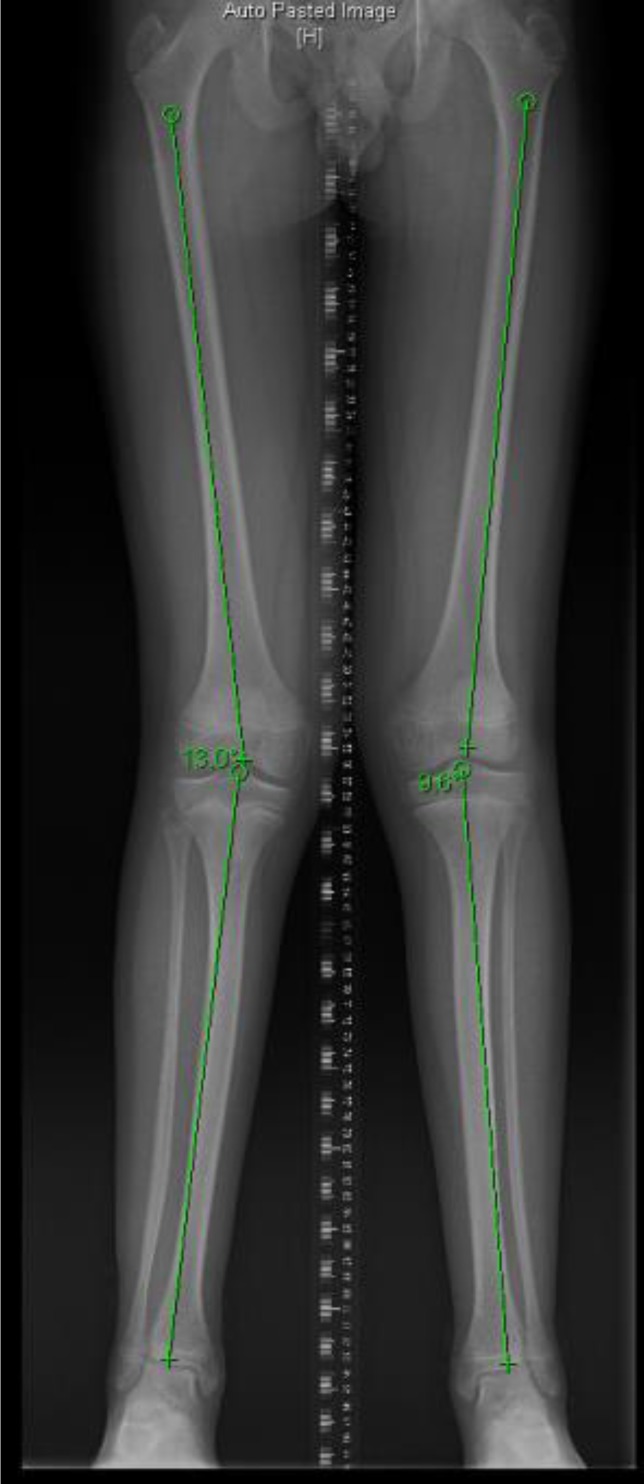
shows asymmetric genu valgum. The right femoro-tibial angle is 13°, the left femoro-tibial angle is 9°.

**Figure 3:**
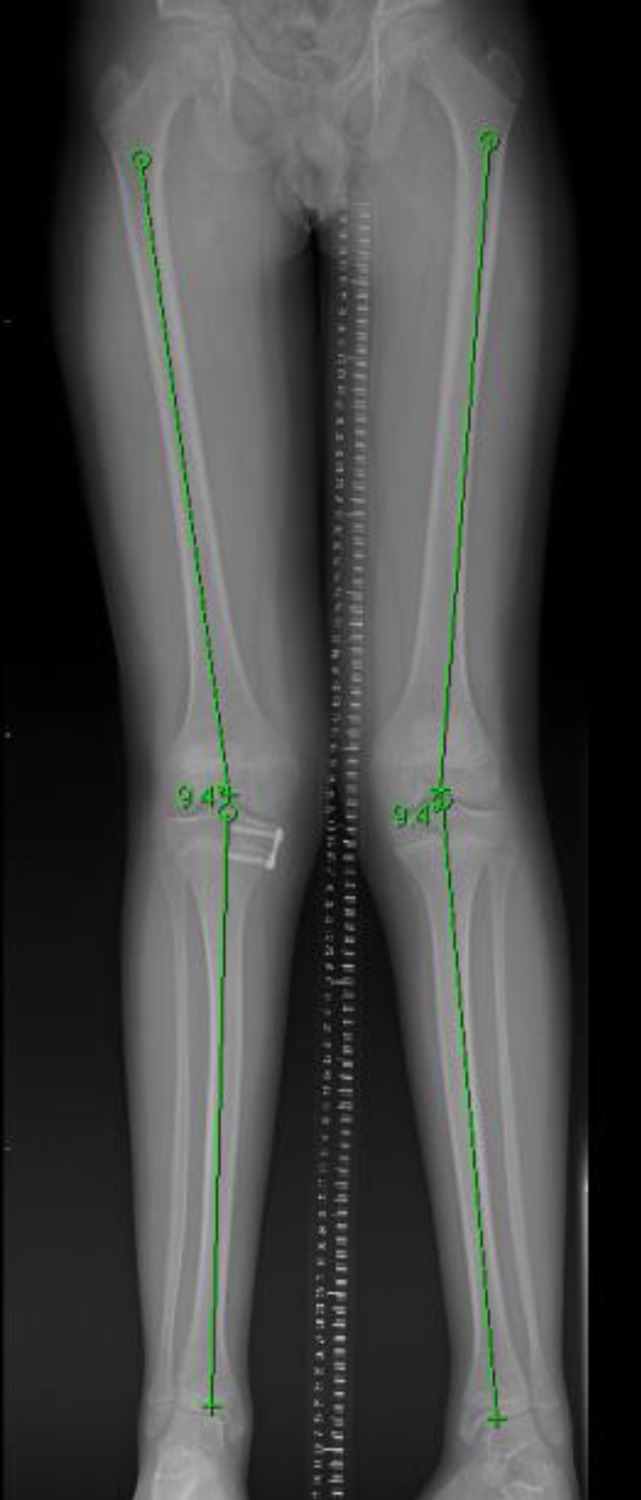
shows restored mechanical axis on both legs after 8-plate insertion. Both femoro-tibial angles are now 9°.

The juxta-physeal infections seem to stimulate the growth plate and cause overgrowth. The direction of the resultant deformity reflects the location of the infection, for example medial involvement leads to valgus malalignment. In our series the, deformities were mild (3-5°). However, in one case the deformity was clinically visible and resulted in mild knee pain, this was successfully rectified by an 8-plate *(shown in Figures 2 and 3)*.

There was one unusual case of a two year old child with a Brodie's abscess in the distal metaphysis of the femur with a sinus draining into the knee joint *(Patient 1 in Table 1)*. No pus was found in the joint, and no organism was identified in this individual. In this patient a small increase in femoral length (<1cm) was noted three years after the infection. The whole physis seemed to have been stimulated. This case is noteworthy as Brodie's abscesses do not commonly form sinuses, secondly the physis of hip, elbow, shoulder and knee joints in very young children are intra-articular.

## Discussion

To our knowledge over-growth after sub-acute osteomyelitis or a Brodie's abscess has not been described elsewhere. In one of the largest case series published to date, Stephens and MacAuley^7^ in 1988 looked at 38 patients with Brodie's abscesses. Patients were followed up until skeletal maturity. According to the authors, none of the patients developed any significant clinical or radiological signs of growth deformity or leg length discrepancy^7^ though two cases of mild leg length discrepancy in their series were noted in Brodie's abscesses close to the physis.

In our case series, all infections that caused a growth effect occurred around the knee. Two of the cases occurred around the distal femoral epiphysis, the fastest growing physis in the body^8^ and two in the proximal tibia. The literature has described metaphyseal tibial lesions as the most common site for Brodie's abscess^9^. It is possible that this overgrowth phenomenon occurs in other growth plates but is not clinically obvious.

Acute osteomyelitis and chronic osteomyelitis are known to cause premature growth plate closure through the formation of bony 'bridges' - columns of new bone that cross the growth plate. It is interesting that the fine slice MRI scan picture shown in Figure 1 shows juxta-physeal osteomyelitis in the metaphysis and epiphysis with a small intra-physeal abscess, yet despite being within the growth plate a bony bridge did not develop.

The cases presented here illustrate the influence that sub-acute osteomyelitis can have on the tightly regulated longitudinal growth of the immature skeleton. Increased vascularity during callus formation has been considered a factor in the overgrowth phenomenon seen after paediatric femur fractures^10^. However acute osteomyelitis is also associated with increased vascularity yet the overgrowth phenomenon has not been observed in this patient group in our institution.

Our study cannot possibly elucidate the way in which the cellular effects of sub-acute osteomyelitis stimulate growth or conversely why acute osteomyelitis does not. Does the abscess lead to overgrowth on the affected site by over-stimulation of the growth plate or does it inhibit regulatory factors within it? It is exciting to consider how a better understanding of this overgrowth phenomenon could unlock potential therapies for physis manipulation.

In our series, deformities were subtle and required long leg standing x-rays to identify them, but in one instance, there was an associated symptom of knee pain. It is interesting to note that pain settled after mechanical axis correction with an 8-plate, and that the deformity has not returned. As the deformity is small it was surprising that it should be clinically significant, we can speculate that the pain was because the deformity was new and asymmetric.

The observation of this overgrowth phenomenon has led us to recommend long-term surveillance using weight-bearing radiographs on children with sub-acute osteomyelitis of the lower limb.

## Figures and Tables

**Table 1:** Summary of the juxta-physeal abscesses that caused subsequent growth deformities

Patient number	Age at presentation	Gender	Surgery	Time to deformity	Organism	Location	Deformity
1	2 years	Male	Yes - arthroscopic washout	36 months	No growth	Distal femur	Long limb
2	1 year	Male	Yes - incision and drainage	20 months	Staph aureus	Distal lateral femur	Genu varum
3	1 year	Male	Yes - incision and drainage	6 months	No growth	Proximal medial tibia	Genu valgum
4	12 years	Male	No	4 months	Staph aureus	Proximal medial tibia	Genu valgum
